# Improved Forceps

**Published:** 1851-01

**Authors:** 


*Improved Forceps.*—We have had placed upon our table a new instrument, or rather an improved one, by Dr. Levi S. Parmly, of New Orleans, for the extracting of wisdom teeth, which, in our opinion, is decidedly the most useful and valuable addition to our extracting instruments which has been made for a long time. The difficulties to be met in the removal of these teeth, are generally found in the roots having grown in a curve backwards with the ramus of the jaw, rendering extraction by the ordinary forceps for lower wisdom teeth, painful, tedious, and sometimes very difficult. It consists in adding to the beak of our ordinary forceps for this purpose, the blades of Physic's forceps, which are forced between the second molar and wisdom tooth, and in closing, the tooth is, at the same time, grasped by the ordinary cylinder-shaped beaks, and thus, with a trifling elevation, the tooth is thrown backwards and upwards, in completely accommodating the curved condition of the roots.

Dr. Levi S. Parmly has also shown us new patterns for scalers, the great merits of which principally reside in the fact, that from their admirable shape, only three are required for removing tartar from a whole set of teeth, under any description of irregularity. We certainly can see the best advantages arising from their general adoption, and take pleasure in recommending them. They can be procured from Francis Arnold, Sharp street, Baltimore.

